# Facile synthesis of reduced graphene oxide–gold nanohybrid for potential use in industrial waste-water treatment

**DOI:** 10.1080/14686996.2016.1201413

**Published:** 2016-07-26

**Authors:** Prasenjit Kar, Samim Sardar, Bo Liu, Monjoy Sreemany, Peter Lemmens, Srabanti Ghosh, Samir Kumar Pal

**Affiliations:** ^a^ Department of Chemical, Biological and Macromolecular Sciences, S. N. Bose National Centre for Basic Sciences, Kolkata, India; ^b^ Institute for Condensed Matter Physics, TU Braunschweig, Braunschweig, Germany; ^c^ Advanced Mechanical and Material Characterization Div., CSIR-Central Glass & Ceramics Research Institute, Kolkata, India; ^d^ Laboratory for Emerging Nanometrology, TU Braunschweig, Braunschweig, Germany

**Keywords:** Graphene oxide, Au-RGO nanohybrid, Rhodamine 123, surface adsorption, NSET, 10. Engineering and structural materials, 104 Carbon and related materials, 212 Surface and interfaces, 503 TEM, STEM, SEM, 505 Optical/Molecular spectroscopy

## Abstract

Here, we report a facile approach, by the photochemical reduction technique, for *in situ* synthesis of Au-reduced graphene oxide (Au-RGO) nanohybrids, which demonstrate excellent adsorption capacities and recyclability for a broad range of dyes. High-resolution transmission electron microscopy (HRTEM), X-ray diffraction (XRD), and X-ray photoelectron spectroscopy (XPS) data confirm the successful synthesis of Au-RGO nanohybrids. The effect of several experimental parameters (temperature and pH) variation can effectively control the dye adsorption capability. Furthermore, kinetic adsorption data reveal that the adsorption process follows a pseudo second-order model. The negative value of Gibbs free energy (ΔG^0^) confirms spontaneity while the positive enthalpy (ΔH^0^) indicates the endothermic nature of the adsorption process. Picosecond resolved fluorescence technique unravels the excited state dynamical processes of dye molecules adsorbed on the Au-RGO surface. Time resolved fluorescence quenching of Rh123 after adsorption on Au-RGO nanohybrids indicates efficient energy transfer from Rh123 to Au nanoparticles. A prototype device has been fabricated using Au-RGO nanohybrids on a syringe filter (pore size: 0.220 μm) and the experimental data indicate efficient removal of dyes from waste water with high recyclability. The application of this nanohybrid may lead to the development of an efficient reusable adsorbent in portable water purification.

## Graphical abstract

**Figure:**
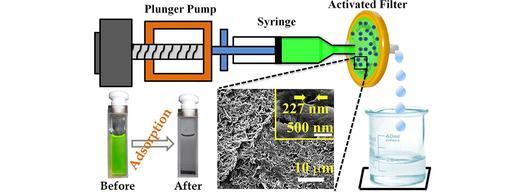

## 1. Introduction

Detoxification of waste water from dyes and pigments is a matter of great concern, since every year 10–15% dyes are wasted during fabrication processes and directly discharged to water causing a pollution of the environment by potential carcinogens.[1] In this regard, efficient adsorption has been considered as the most preferred way for the easy and efficient removal of toxic dyes from waste water.[2–5] However, most of the common adsorbents including activated carbon, rice husk, zeolites and mesoporous silica suffer from low adsorption capacities, poor selectivity and an unsatisfactory recyclability.[6–8] Moreover, a number of advanced adsorbents have been reported using low-cost carbon and inorganic origin, such as carbon nanotubes, graphite, fullerene, nanostructured metal oxides, and porous boron nitride nanosheets, to circumvent this emerging problem.[8–10] However, the engineered carbon materials have a limited adsorption capacity and often are toxic to the environment.[11] Therefore, a versatile adsorbent with enhanced efficiency would be highly desirable for economical water purification. Recently, graphene consisting of single layer of carbon atoms with a honeycomb two-dimensional (2D) lattice crystal structure has been recognized as a promising material in various fields, such as solar energy harvesting, drug delivery, nanoelectronics, sensors, H_2_ production and water purification.[12–14] Li et al. [15] have synthesized stable aqueous dispersions of graphene sheets by photophysical reduction of graphene oxide (GO) without using any chemical additives.[15] Recent activities focused on graphene or GO as an attractive adsorbent material due to its high surface area, negative surface charge and highly mobile π electrons. Its electronic configuration can easily undergo electrostatic and π–π interactions with dyes containing a π-electron rich structure. Nonetheless, the van der Waals and π–π interactions between individual graphene layers significantly decrease their hydrophilic character and consequently lead to their irreversible agglomeration.[16]

Nanohybrids have attracted tremendous attention due to their potential functionality for tailored applications.[17−21] Graphene based composites are novel hybrid materials where inorganic particles are synthesized on graphene sheets which act as two dimensional substrate to prevent nanoparticles (NPs) aggregation.[22,23] The nanohybrid displays fascinating physicochemical properties as a consequence of the intimate dispersion or mixing of the both components which are useful in catalysis, energy storage, water purification and drug delivery.[14,24,25] Furthermore, presence of metal NPs act as a spacer within the graphene sheet, preventing agglomeration and restacking of the graphene layer during the reduction process; this is used for adsorption and other catalytic applications. Liu *et al.* [26] showed that attachment of carbon coated Fe_3_O_4_ NPs on GO could be used as a promising applied material for toxic dye removal and could be recycled.[26] The synthesis of Au-RGO nanohybrid by using hydrothermal, chemical reduction, physical vapor deposition and electrostatic interaction is reported in the literature.[27–30] However, such techniques require multi-step fabricating processes, involve toxic chemicals and thereby reduce the efficiency of the Au-reduced GO (Au-RGO) hybrid. Therefore, it is essential to develop a facile approach for synthesis of Au-RGO nanohybrids with improved adsorption efficiency. One-pot reductant-free nanohybrid synthesis by photo-reduction is one of the aims of our present work. Up to now only very few reports on the synthesis of Au-RGO nanohybrids by photochemical reduction exist. Wang *et al.* [31] synthesized Au-RGO nanohybrids by using UV illumination and N-hexadecyltrimethylammonium chloride (CTAC) used as a surfactant to protect NPs from agglomeration.[31] Uses of surfactants are not compatible with graphene and sometimes interfere between graphene and metal NPs interface. Moreover, details of adsorption studies by Au-RGO nanohybrids and their correlation with ultrafast electron transfer dynamics by spectroscopy are very sparse in the existing literature and also one of the motives of our present work.

Herein, we have developed a facile approach for the synthesis of Au-RGO nanohybrid via the photochemical reduction technique. Adsorption capacity of the synthesized nanohybrid has been studied for a wide range of dyes. We further studied the effect of pH and temperature for the adsorption process. Details of adsorption kinetics and thermodynamic parameters are also investigated using Rhodamine 123 (Rh123) as a model dye. Picosecond resolved fluorescence technique has been employed to investigate the proximity of the dye after adsorption on an Au-RGO surface. Nanometal surface energy transfer is found be effective between Rh123 and Au NPs after adsorption of Rh123 on Au-RGO nanohybrid. For potential application, we have developed a prototype device by loading Au-RGO nanohybrid in a syringe filter mesh (pore size 0.220 μm) and studied adsorption efficiency and recyclability of the device using Rh123 and crystal violet as model dyes.

## 2. Experimental section

Graphite powder, gold acetate, Coumarin-500, crystal violet, eosin, and Rhodamine 123 were purchased from Sigma. Methylene blue was purchased from Carlo Erba (Val-de-Reuil, France). All other chemicals used in this study were of analytical grade.

### 2.1. Synthesis of Au-RGO nanohybrid

At first GO was synthesized from graphite powder according to our previously published work.[32] The GO (1 mg ml^–1^) was then dispersed in 2-propanol and exfoliation takes place in an ultrasonic bath for 20 min. Then 3 mM of gold (III) acetate was dissolved in GO solution followed by deoxygenation under a N_2_ flow. The GO sample was then exposed to UV-irradiation at room temperature for 5 h. Then the as-synthesized Au-RGO nanohybrid was dried in an oven. Reduced GO was obtained using similar procedure without using metal salts.

### 2.2. Adsorption experiments

Methylene blue, eosin, Rhodamine 123, coumarin and crystal violet stock solutions were prepared by dissolving in water (1 mg ml^–1^). The desired dye concentration used in this study was 10 μM, obtained by diluting the stock solution in accurate proportions. The pH of the solution was maintained by using 0.01 N HCl and NaOH solution. The detailed adsorption kinetics of Rh123 on the Au-RGO nanohybrid was monitored based on absorption spectroscopy. To probe adsorption behavior, different concentrations of Rh123 solution (total volume 2 ml) were prepared from stock solution with proper dilution using deionized water (DI). In each case the equal amount of adsorbent (0.5 mg ml^–1^) were added. Therefore, the adsorption capacity, *q*_*e*_ (μmol g^–1^) can be calculated by using the following equation:

(1) $$q_{e}=\frac{V}{m}\left(C_{0}-C_{t}\right),$$

where C_0_ is the initial concentration of dye (μM), C_t_ is the concentration at time t (μM), V is the volume of the suspension (L) and m is the mass of the adsorbent (g). pH dependency studies were performed at different pH (2, 4, 6, 8 and 10) by adjusting with 1 N NaOH or HCl solution. In order to find out the influence of temperature on adsorption kinetics of Rh123 on Au-RGO nanohybrid, experiments were carried out at five different temperatures (20, 30, 40, 50 and 60°C) at pH 6.

### 2.3. Characterization techniques

Transmission electron microscopy (TEM) grids were performed by addition of a diluted drop of Au-RGO nanohybrid samples to carbon-coated copper grids using an FEI Technai S-Twin instrument (FEI, Hillsboro, OR, USA) operated at 200 kV. Field emission scanning electron microscopy (FESEM, QUANTA FEG 250) investigations were performed by applying a diluted drop of Au-RGO nanohybrid on a silicon wafer. X-ray diffraction (XRD) of the patterns were obtained in the 2θ range from 20° to 80° by employing a scanning rate of 0.02° S^–1^ using a PANalytical XPERTPRO diffractometer equipped with Cu Kα radiation (at 40 mA and 40 kV). Raman scattering experiments have been carried out by exploiting a micro-Raman setup (Horiba LabRAM [Horiba, Edison, NJ, USA]) with excitation line 532 nm at room temperature. Raman spectra of all the samples have been recorded at room temperature in the frequency range 150–3400 cm^–1^. Zeta potential measurements were done by DLS-nanoZS, Zeta sizer, Nanoseries (Malvern Instruments, Malvern, UK). For X-ray photoelectron spectroscopy (XPS) study, RGO and Au-RGO samples were prepared in thin film form on Si-substrates (SiO_2_/Si) and the analysis was carried out in a PHI 5000 VersaProbe II spectrophotometer (Physical Electronics Inc., Eden Prairie, MN, USA) using a monochromatized Al K_α_ (~1486.6 eV) X-ray beam of size ~ 100 μm. Prior to XPS analysis, sample surfaces were sputtered with a 2 kV rastered Ar^+^ ion beam for one minute to clean the surface. During XPS measurements, dual beam charge neutralization system was operated in order to neutralize the generated static charges on the sample surface. Recorded high resolution C 1s photoelectron spectra were resolved into their respective Gaussian fits after removal of background intensity. Absorption spectra of samples were monitored by Shimadzu UV-2600 spectrophotometer (Shimadzu Corporation, Kyoto, Japan), while steady state fluorescence spectra of samples were monitored using JobinYvon Fluorolog fluorometer (Horiba, Edison, NJ, USA). Time resolved optical studies were performed by following the methodology as described in our earlier work.[33] The fluorescence quantum yield of Rh123 in water was reported 0.89[34]. The Förster resonance energy transfer (FRET) distance between the donor and acceptor (r) was calculated using the following equation:

(2) $$r^{6}=\left(1-E\right)/E,$$

where E is the efficient of energy transfer and following the procedure published earlier.[35]

The nanosurface energy transfer (NSET) model become useful when the rate of surface energy transfer is expected to 1/d^4^ distance dependent. The d_0_ value was calculated using NSET equation as follows:

(3) $$d_{0}=\left(0.225\frac{c^{3}\phi_{dye}}{\omega_{dye}^{2}\omega_{F}\kappa_{F}}\right),$$

where *ϕ*_*dye*_ is the quantum yield of dye, C is the speed of light, *ω*_*dye*_ is the angular frequency of dye, *ω*_*F*_ is the angular frequency of bulk gold, *κ*_*F*_ is the rate of energy transfer and details of the procedure are discussed in our previous studies.[36]

## 3. Results and discussion

**Figure 1.**
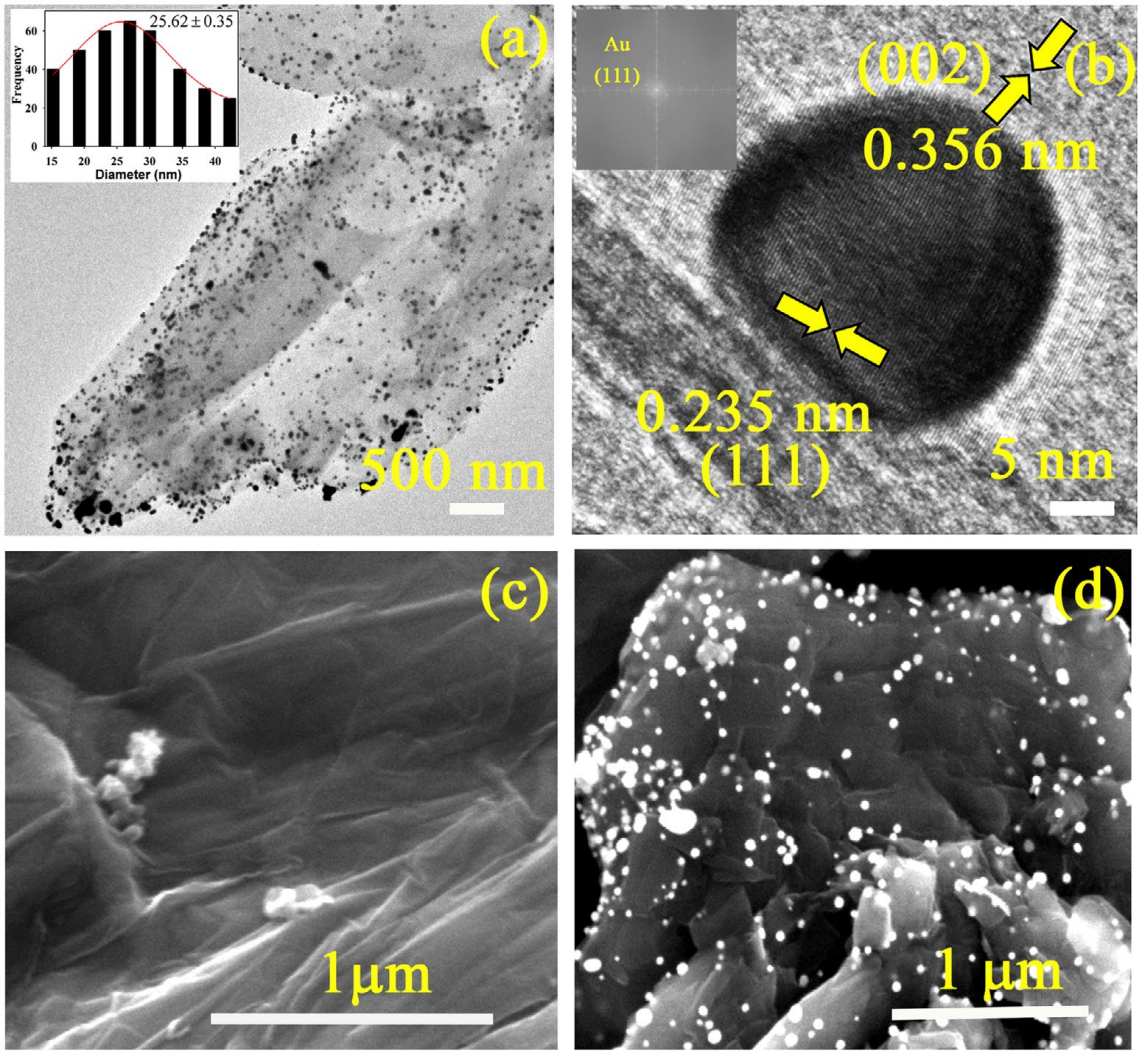
(a) TEM image of the as synthesized Au-RGO nanohybrids (inset shows size distribution of Au NPs). (b) HRTEM image of a Au NP attached to the RGO (inset shows FFT pattern of Au-RGO). SEM images of (c) RGO and (d) Au-RGO.

The formation of Au-RGO nanohybrids has been confirmed by TEM. From Figure 1(a), the nanosheet like morphology of RGO is clearly visible along with uniformly high coverage of Au NPs. The average particle size of Au NPs anchored on the RGO nanosheet is found to be 25.62±0.35 nm. The HRTEM image and fast Fourier transform (FFT) pattern of the Au-RGO nanohybrid (Figure 1(b)) demonstrate high crystallinity of the Au NPs as well as the RGO nanosheet. The inter-planar distance between the fringes is found to 0.235 nm which correspond to the (111) plane of Au NPs.[37] However, lattice spacing of 0.356 nm for RGO corresponds to (002) planes.[38] Formation of nanohybrids was further confirmed by SEM. Figure 1(c) shows an SEM image of an RGO having typical wrinkled and paper-like sheet morphology. Figure 1(d) illustrates an SEM image of Au-RGO nanohybrid, and reveals that the nanoparticles are well dispersed on graphene nanosheets which is consistent with TEM image.

**Figure 2.**
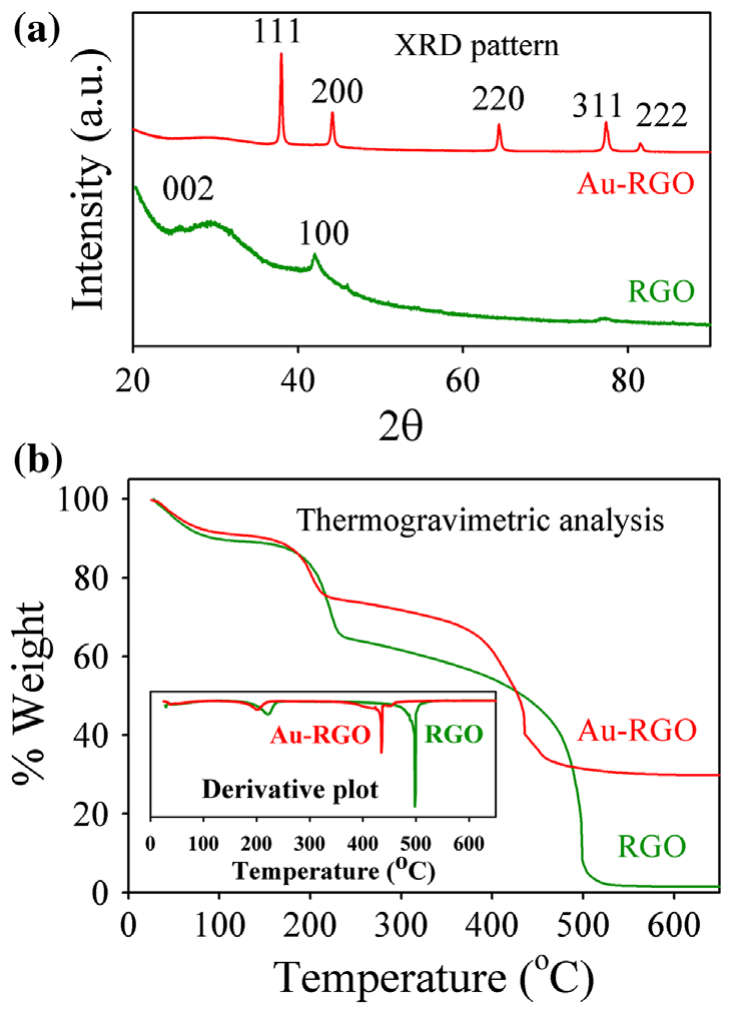
(a) XRD patterns of Au-RGO and RGO. (b) TGA curves of Au-RGO and RGO.

The presence of Au NPs on RGO nanosheets was further characterized by X-ray diffraction (XRD). From Figure 2(a) the diffraction peaks can be indexed as the (111), (200), (220), (311) and (222) reflections of the face-centered cubic (fcc) structure of Au (JCPDS 04-0784).[39] Additionally, exfoliation behavior of RGO in presence of Au NPs can be explained by XRD analysis.[40] After photochemical reduction of GO, the broad diffraction peak at 24º arises due to partial restacking of some GO layers.[40] However, a broad peak at 24º disappears in Au-RGO due to Au NPs effectively prevented the restacking of the GO layer. Thus, the XRD pattern indicates that Au NPs successfully incorporated between RGO layers and act as a spacer between two layers to prevent restacking. Thermogravimetry analysis (TGA) curves of both RGO and Au-RGO shows an obvious weight loss between 160 and 220°C which is attributed to pyrolysis of the labile oxygen-containing functional groups, yielding CO, CO_2_ and steam [41] as shown in Figure 2(b). RGO shows a complete decomposition of carbon at about 500°C. In contrast, the Au-RGO nanohybrid shows a lower temperature of about 420°C, to fully decompose the carbon of Au-RGO. A residual mass of about 30% indicates loading of Au NPs in the Au-RGO nanohybrid. A lower thermal decomposition temperature for Au-RGO compared to RGO, due to presence of Au NPs, helps RGO in exfoliation, thereby increasing the interlayer spacing and porosity of the hybrid.

**Figure 3.**
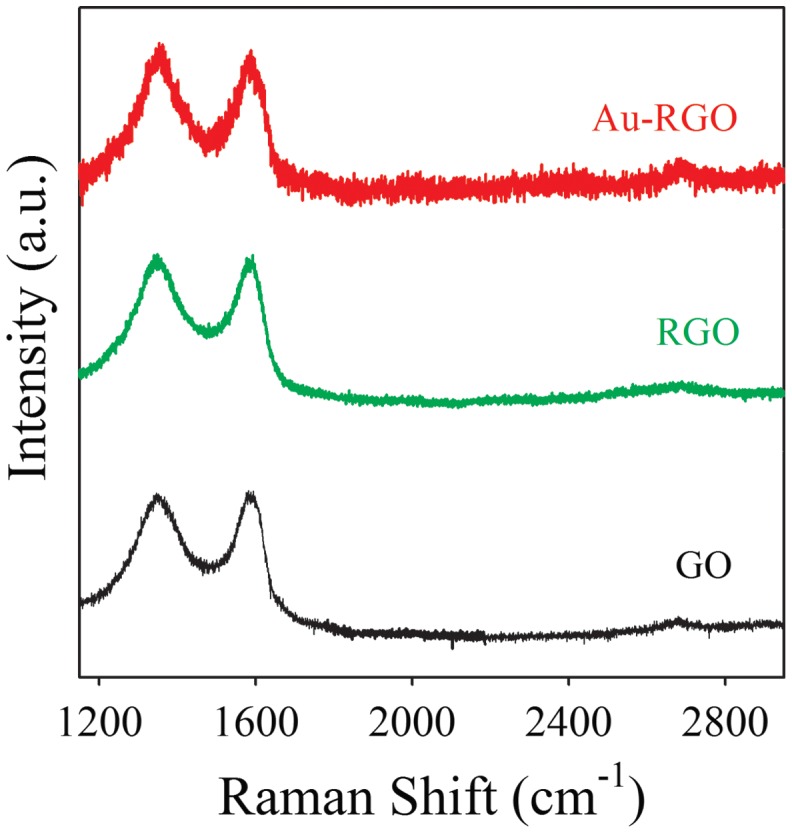
Raman spectra of graphene oxide (GO) and Au-reduced graphene oxide (Au-RGO) nanohybrids. The baselines of the spectra are shifted for clarity.

In order to further investigate the structural and electronic properties of Au-RGO nanohybrids, Raman spectroscopy has been used as a tool. Raman spectra of GO, RGO and Au-RGO are shown in Figure 3. GO exhibits two characteristic peaks located at 1340 and 1580 cm^–1^, which can be attributed to D and G bands, respectively, from GO. The D bands correspond to a defect induced in plane A_1g_ zone-edge mode and G band is attributed to E_2g_ mode.[31] After photoirradiation, RGO and Au-RGO show an increased intensity ratio (I_D_/I_G_) of 1.01 and 1.12, respectively, in comparison to GO (0.98), indicating a decrease in size of the sp^2^ domains.[42] Here, after photoirradiation, a broad 2D band for the Au-RGO nanohybrid located at 2632 cm^–1^, indicating the formation of layer graphene nanosheet, is present in the nanohybrid.[31]

**Figure 4.**
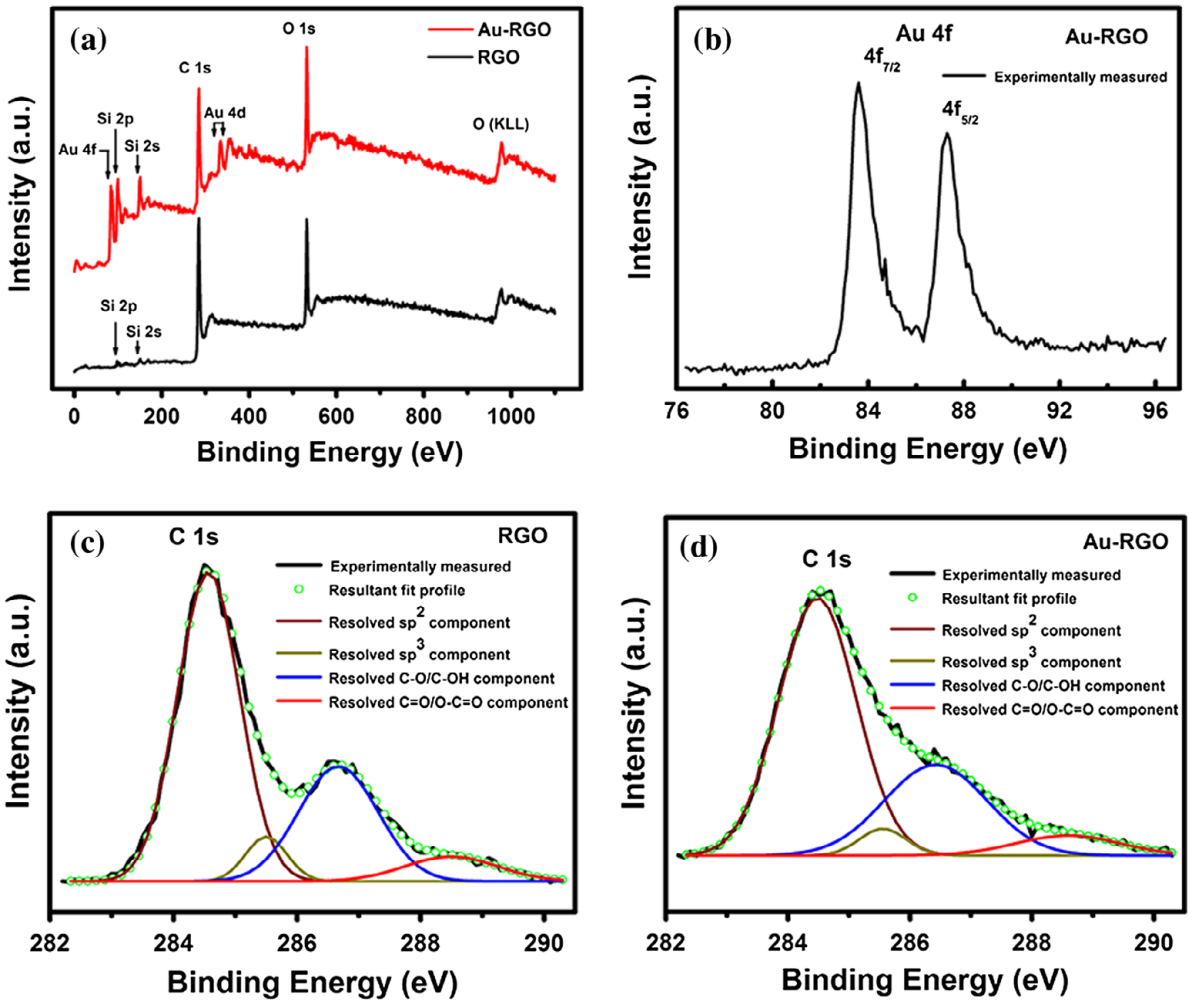
Survey and high resolution XPS spectra of RGO and Au-RGO: (a) survey scans of RGO and Au-RGO. (b) high resolution Au 4f spectrum of Au-RGO. (c) high resolution C 1s spectrum and resolved components of RGO. (d) high resolution C 1s spectrum and resolved components of Au-RGO.

The XPS technique becomes useful in order to further investigate the structural evolution of the as-synthesized Au-RGO nanohybrid. Survey-scan XPS spectra obtained from RGO and Au-RGO are shown in Figure 4(a). Due to the deposition of a thin layer of the materials on the Si substrate and the takeoff angles used for XPS analysis, the signature of silicon (Si) signal arising from the Si-substrate underneath can be detected in the survey scans of both the samples. It is evident from Figure 4(a) that carbon (C) and oxygen (O) are the primary constituents of RGO while Au-RGO is composed of C, O and gold (Au). Figure 4(b) shows the high-resolution Au 4f spectrum recorded from Au-RGO sample where Au 4f_7/2_ and Au 4f_5/2_ spin-orbit doublets are observed to be located at ~83.6 and 87.3 eV respectively in the binding energy scale. Herein, the chemical nature of gold within Au-RGO has been attributed as metallic-Au (Au^0^) which is consistent with previous literature.[43–45] The observed small negative shift (~0.4 eV) in the position of Au 4f spin-orbit doublet with respect to that of the standard referencing positions of metallic-Au (Au^0^: Au 4f_7/2_ ~ 84.0 eV and Au 4f_5/2_ ~87.7 eV) suggests that a strong interaction exists between Au and RGO framework.[44, 45] High resolution C 1s spectra of RGO and Au-RGO can be resolved into four Gaussian components. Peaks centered at binding energy positions of ~284.5, 285.5, 286.6 and 288.5 eV have been assigned to sp^2^ (C=C), sp^3^ (C-C, C-H), epoxy and/or hydroxyl (C-O/C-OH) and carbonyl and/or carboxyl (C=O/O-C=O) functional groups respectively (Figure 4(c) and 4(d)).[46,47] As shown in Figure 4(c) and 4(d), an excellent match between the experimentally measured C 1s profile (represented by a solid black line) and the resultant fit profile (represented by green colored hollow circles) signifies the merit of the peak fitting protocol undertaken to resolve the C 1s profiles into their respective components. However, the concentrations of these functional groups are found to vary depending on the reduction protocol employed to reduce the GO. Compositions of RGO and Au-RGO as obtained from XPS analysis are listed in Table 1. The degree of reduction of RGO can be determined by the abundance of sp^2^ hybridized carbon relative to other oxygenated carbon groups (e.g. epoxy, hydroxyl, carboxyl, and carbonyl) in it. In this regard, the degree of reduction of Au-RGO is found to be slightly higher than that of RGO sample (Table 1). Also, the concentration of sp^3^-C in RGO is estimated to be higher than Au-RGO (Table 1).

**Table 1.** Compositions of RGO and Au-RGO as obtained from XPS analysis.

Sample	Atomic ratio	Concentration (%)			
	(Au/C)	sp^2^ -C	sp^3^ -C	C-O/C-OH	C=O/O-C=O
		(C=C)	(C-C/C-H)	(epoxy/hydroxyl)	
Au-RGO	0.03	61.3	3.8	28.6	6.3
RGO	–	59.2	5.7	28.1	7.0

**Figure 5.**
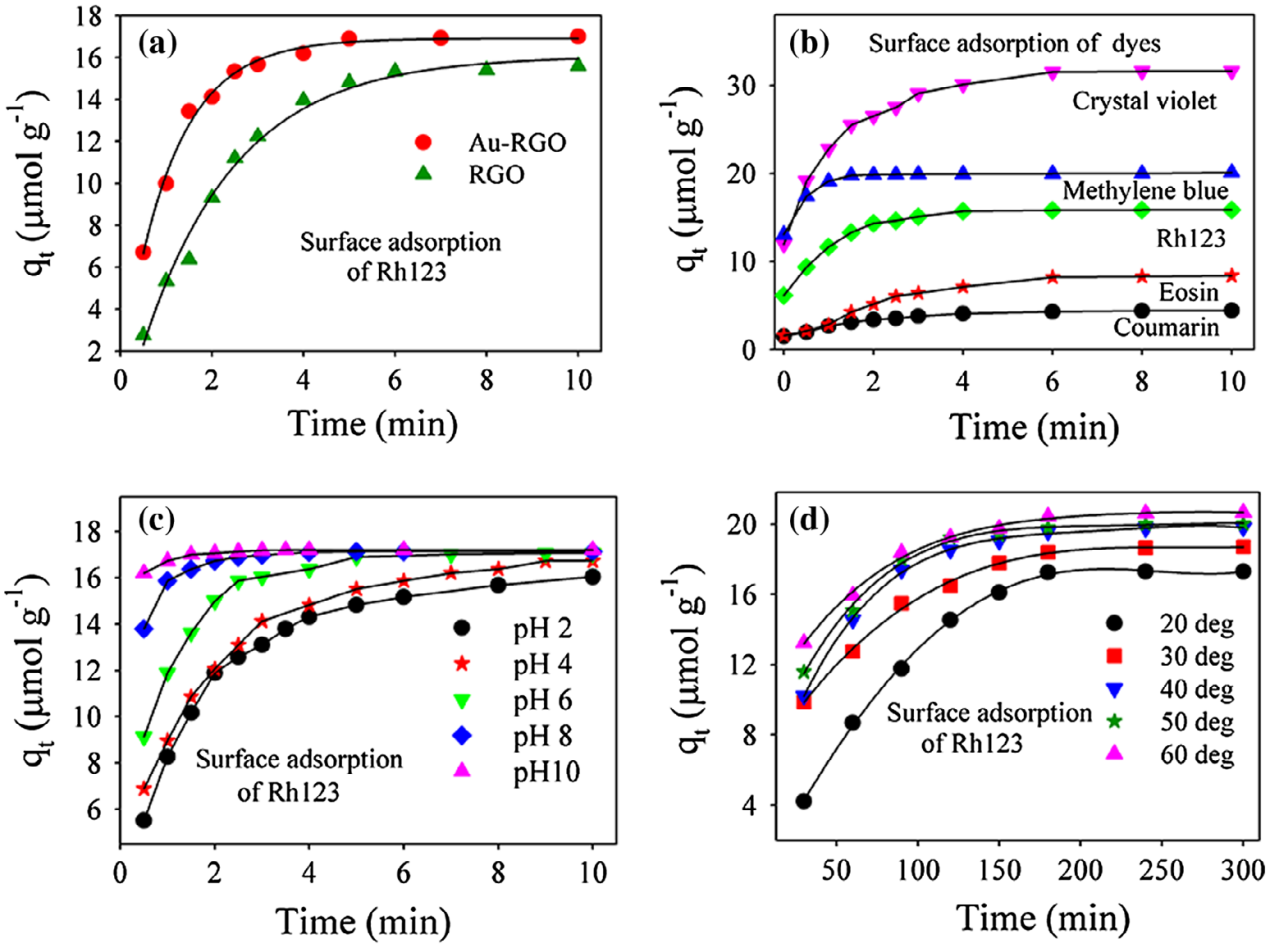
Effect of adsorption rate: (a) Au-RGO versus RGO. (b) different dyes. (c) different pH; and (d) different temperatures conditions (solid lines are guide to the eyes).

Adsorption behaviors of Au-RGO nanohybrid were studied with different dyes at varied pH and temperature values, as shown in Figure 5. From Figure 5(a), it is observed that the adsorption capacity of Au-RGO is higher compared to the RGO nanosheet. This observation could indicate that the presence of Au NPs decorated on an RGO sheet enhances the exfoliation of RGO by acting as a spacer. Thus, an increase in effective surface area facilitates enhanced dye adsorption. Adsorption capacities of the dyes by the Au-RGO nanohybrid are shown in Figure 5(b). It is observed that the Au-RGO nanohybrid is highly efficient for removal of cationic dyes in comparison to anionic and neutral dyes. The results indicate that adsorption capacity for crystal violet, methylene blue, Rh123, eosin, and coumarin were 31, 19, 17, 7 and 5 μmol g^–1^ respectively at pH 6. The adsorption capacity of the Au-RGO nanohybrid is comparatively higher than other graphene derivatives [22, 48–50] as shown in Table 2. The higher adsorption capacity of Au-RGO nanohybrid towards cationic dyes suggests strong π–π and electronic interaction between Au-RGO and cationic dyes. This is in conformity to that observed in the case of negative surface charge (–35 mV) of the Au-RGO nanohybrid.

**Table 2.** Comparison of the maximum adsorption capability of various adsorbents for rhodamine.

Adsorbents	Capacity (mg g^–1^)	Contact time (min)	Reference
Graphene oxide	29	180	[49]
Graphitic N-doped carbon nanoparticles-decorated carbon flake	13.7	60	[48]
Hybrid of reduced graphene oxide-Fe_3_O_4_ nanoparticles	50	720	[22]
Magnetic-reduced graphene oxide nanocomposite	13.5	120	[47]
Au-RGO nanohybrid	34.3	10	Present work

The effect of pH on adsorption has been studied over a wide range as shown in Figure 5(c). It is observed that an increase in adsorption efficiency from 15.3 to 17.2 μmol g^–1^ occurs with an increase in pH from 2 to 10. The higher adsorption capacity of Rh123 at high pH may be due to increased negative charge at the surface of adsorbents, which may facilitate more Rh123 dye coming into contact with the Au-RGO surface. The reason for cationic dye adsorption with increase in pH is due to an increase in ionic interaction in addition to π–π stacking between the more negatively charged GO surface and cationic dye.[51] In order to find out the effect of temperature on adsorption rate, the adsorption experiments were conducted at five different temperatures (20, 30, 40, 50 and 60°C) at pH 6. It is observed that adsorption capacity of Rh123 on the Au-RGO nanohybrid increases from 17.36 to 20.60 μmol g^–1^ as the temperature increases from 293 to 333 K. The higher adsorption capacity of Au-RGO nanohybrids with increase in temperature indicates that the thermal energy allows the dye molecules to come into contact with the internal pores of the RGO nanosheet, thereby increasing the adsorption capacity.

**Figure 6.**
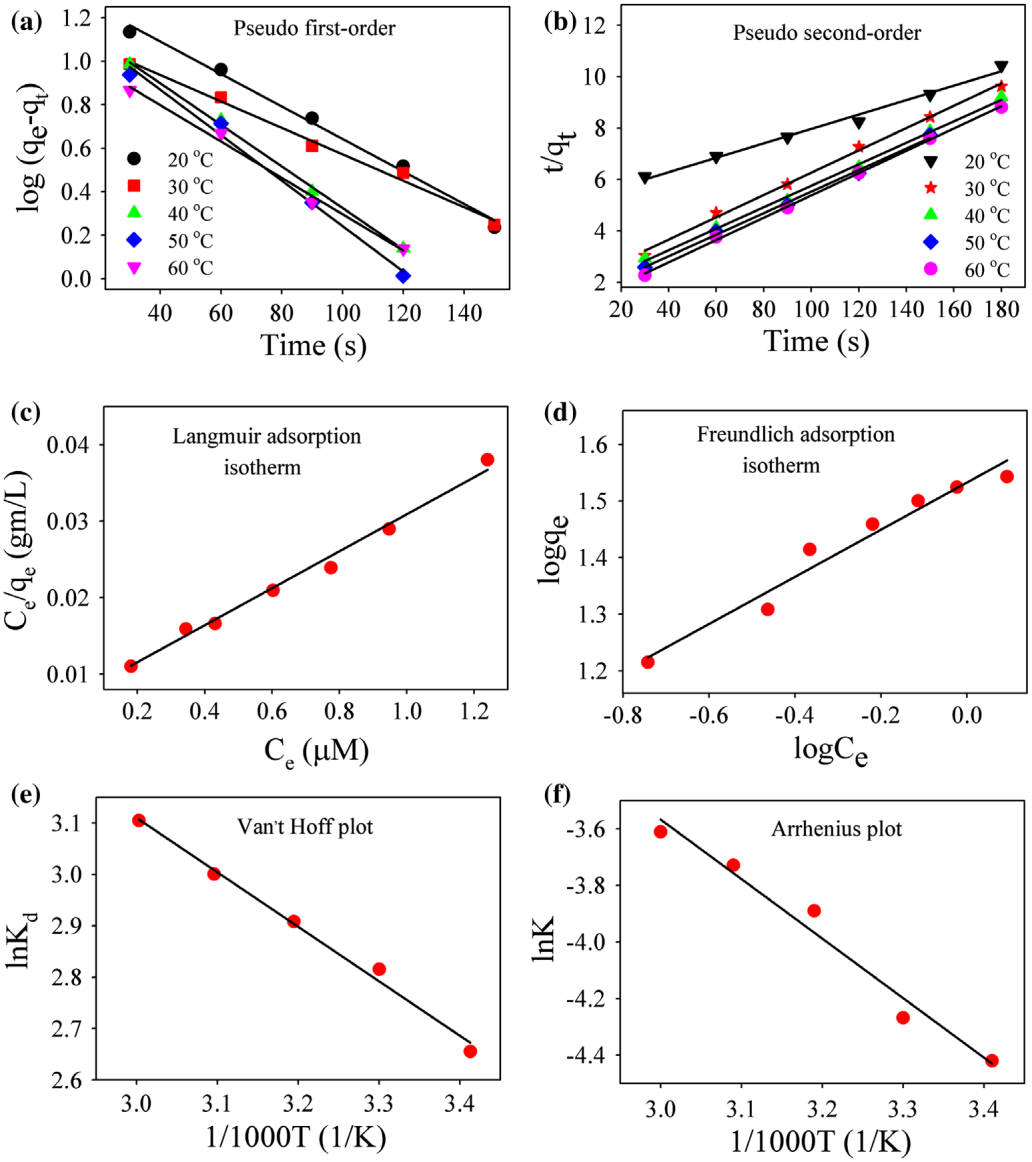
Plot of (a) pseudo first-order model. (b) pseudo second-order model. (c) Langmuir adsorption isotherm and (d) Freundlich adsorption isotherm for Rh123 adsorption. (e) Van’t Hoff plot and (f) Arrhenius plot for Rh123 adsorption.

Kinetics of adsorption of Rh 123 on an Au-RGO nanohybrid has been examined by the pseudo first-order model and pseudo second-order model as shown in Figure 6(a) and 6(b). The models are described in the supporting information. For the pseudo first-order model and the pseudo second-order model, the values of rate constant and adsorption efficiency are presented in Table 3. From the table, it is evident that correlation coefficients (R^2^) for pseudo second order are better than that of the pseudo first-order model. Therefore, adsorption of Rh123 on Au-RGO nanohybrids follows the pseudo second-order model more correctly than the pseudo first-order model.[52, 53] The plot of C_e_/q_e_ versus C_e_ gives a straight line indicating that adsorption of dye follows the Langmuir adsorption isotherm model as shown in Figure 6(c). The values of Q_0_ and K_L_ calculated from the slope and intercept are shown in Table 4. The plot of logq_e_ versus logC_e_ gives a straight line, indicating that adsorption of dye follows the Freundlich adsorption isotherm model as shown in Figure 6(d). The values of n and K_f_ calculated from the slope and intercept are shown in Table 4. The R^2^ value for Langmuir adsorption isotherm is high compared to the Freundlich adsorption isotherm, which indicates adsorption of Rh123 on the Au-RGO nanohybrid preferably follow the Langmuir adsorption isotherm. These results are in good agreement with reported results.[26, 54] The calculated thermodynamic parameters are summarized in Table 5. The negative free energy values at different temperatures suggest the feasibility of the process for the adsorption of Rh123 onto Au-RGO nanohybrids. The calculated positive ∆H^0^ value (8.72 kJ mol^–1^) indicates the endothermic nature of the process, whereas positive entropy change (0.05 kJ mol^–1^ K^–1^) indicates the good affinity of Rh123 toward the Au-RGO nanohybrid and the increased randomness at the Au-RGO nanohybrid−water interface during the adsorption process.[55–57] The activation energy (E_a_) gives an idea about the nature of the adsorption process. Therefore the value of activation energy (E_a_) for the adsorption of Rh123 onto Au-RGO nanohybrid can be calculated by using the Arrhenius equation:

(4) $$\ln\text{K}=lnA-E_{a}/RT,$$

**Table 3.** Kinetic model parameters for the adsorption of Rh123 onto Au-RGO nanohybrids.

Model	Parameters	Temperature (°C)				
		20	30	40	50	60
Pseudo first-order	K_1_ (s^–1^)	0.017	0.014	0.022	0.024	0.019
	R^2^	0.990	0.989	0.996	0.986	0.987
Pseudo second-order	K_2_ (μM^–1^ s^–1^)	1.52×10^–4^	9.78×10^–4^	1.10×10^–3^	1.30×10^–3^	1.81×10^–3^
	R^2^	0.985	0.995	0.997	0.995	0.998

**Table 4.** Langmuir and Freundlich constants for adsorption of Rh123 onto Au-RGO nanohybrids.

Temperature (K)	pH	Langmuir constants	
303	6	Q_o_ (μmol g^−1^)	K_L_ (μmol g^–1^)
		38.71	0.227
		Freundlich constants	
		n	K_F_ (μmol g^–1^)
		2.39	34.1

**Table 5.** Thermodynamic parameters for adsorption of Rhodamine 123 onto Au-RGO nanohybrids.

Temperature (°C)	ΔG^°^ (kJ mol^–1^)	ΔH^°^ (kJ mol^–1^)	ΔS^°^ (kJ mol^–1^ K^–1^)
20	−6.46	8.33	0.05
30	−7.07		
40	−7.54		
50	−8.05		
60	−8.58		

where A is the Arrhenius pre-exponential factor, R is the gas constant and E_a_ denotes the energy of activation during adsorption process. From the plot of ln K versus 1/T (as shown in Figure 6(f)), the activation energy was found to be 17.45 kJ mol^–1^. A low activation energy (< 40 kJ mol^–1^) implies physical adsorption process whereas a relatively high activation energy (> 40 kJ mol^–1^) corresponds to the chemical adsorption process.[53] Hence, low activation energy indicates that the adsorption of Rh123 on Au-RGO nanosheet occurs by physical adsorption process which is consistent with previous literature.[53, 54]

**Figure 7.**
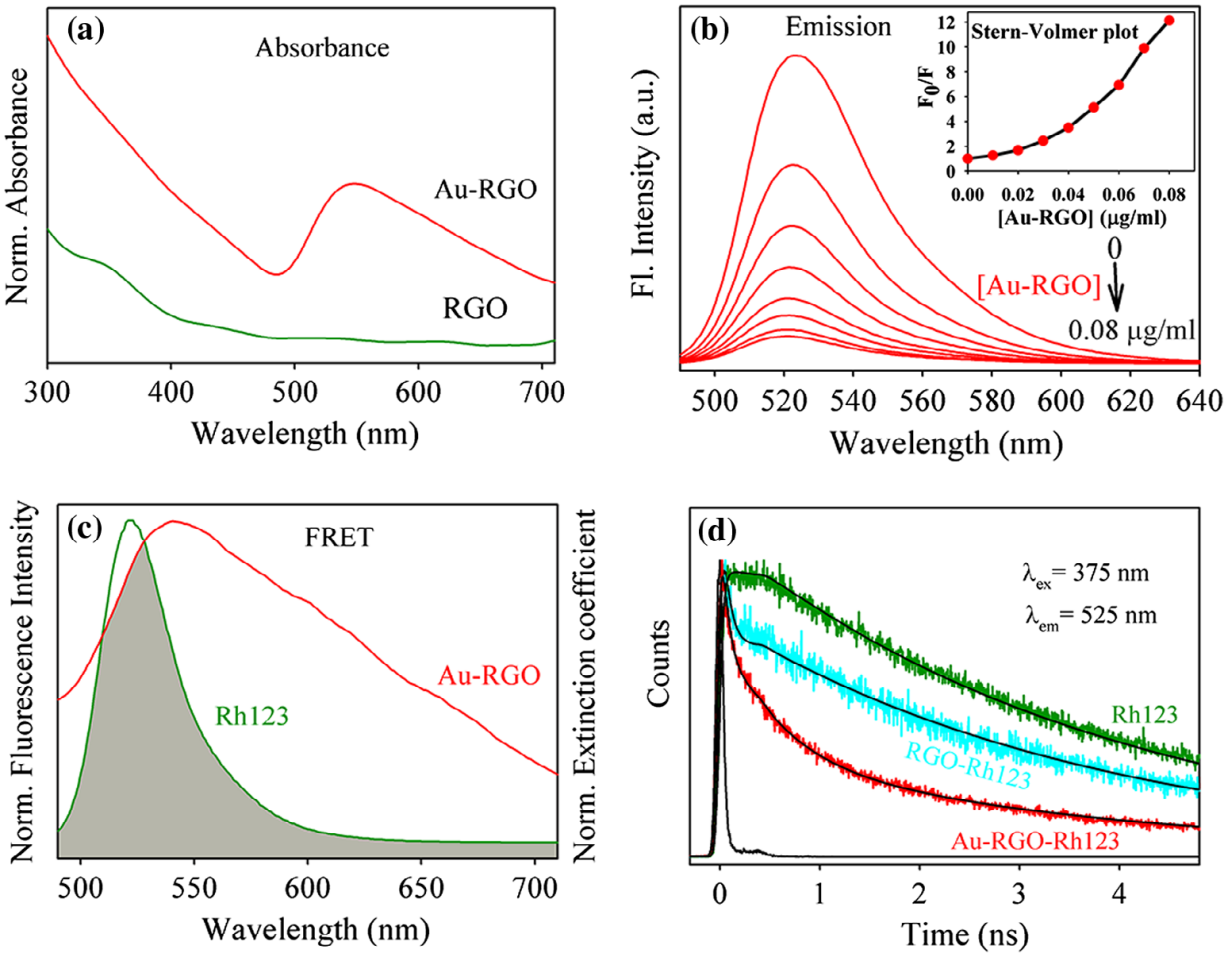
(a) UV-vis absorption plot for Au-RGO and RGO. (b) The effect of Au-RGO hybrid on the fluorescence intensity of Rh123 (inset shows Stern–Volmer plot). (c) The overlap integral of Au-RGO absorbance and Rh123 emission. (d) Fluorescence decay profiles of Rh123, RGO-Rh123 and Au-RGO-Rh123.

Figure 7(a) shows the UV-vis absorption spectra of RGO and Au-RGO. The absorption spectrum of RGO shows a peak at 306 nm indicating incomplete reduction of GO.[58] On the other hand, the absorption spectrum of Au-RGO shows an additional peak at 540 nm due to surface plasmon resonance of gold NPs, indicating decoration of Au NPs on the RGO nanosheet as shown in Figure 1. A monotonous decrease of in the fluorescence intensity of Rh123 upon adsorption at the Au-RGO nanohybrid is shown in Figure 7(b). The plot of relative intensity (F_0_/F; where F_0_ and F are the fluorescence intensity of Rh123 without and with Au-RGO respectively) with various Au-RGO concentrations is shown in the inset of Figure 7(b). A deviation from linearity in the increase of F_0_/F with gradual increase in the Au-RGO concentration indicates that both static and dynamic quenching is associated in the quenching process.[59] The quenching of Rh123 followed by surface adsorption at the Au-RGO surface can be rationalized in the following ways. Firstly, the remarkable spectral overlap (J(λ)=2.04X10^20^ M^–1^ cm^–1^ nm^4^) of the Au-RGO absorption spectrum with that of the emission spectrum of Rh123 reveals possibility of (FRET) or (NSET) from Rh123 to the surface of Au-decorated RGO. A significant shortening of fluorescence lifetime of Rh123 on the Au NP decorated RGO compared to that in bulk water is shown in Figure 7(d) and Table 6. Following FRET strategy[60] we have estimated the donor (Rh123) acceptor (surface of Au NPs) distance is found to be 36.3 nm, which is beyond the probing limit of FRET (~10 nm) and much higher than the diameter of the Au NPs as revealed from the TEM micrograph (Figure 1). Thus FRET is not expected to be the quenching mechanism of the probe Rh123 at the surface of the Au NPs in the nanosheet of RGO. The donor–acceptor distance following NSET strategy is 7.4 nm, which indicates proximity of the dye Rh123 at the Au NPs (average diameter of ~25 nm). It should be noted that the fluorescence quenching of Rh123 could be associated with the photoinduced electron transfer from the dye to the RGO surface.[61] As shown in Figure 7(d) and Table 6, a faster time component in the fluorescence decay of Rh123 at the RGO surface in absence of Au NPs is eminent and consistent with photoinduced electron transfer.[62]

**Table 6.** Lifetimes of picosecond time-resolved fluorescence transients of Rh123, Rh123-RGO, and Rh123-Au-RGO, detected at various fluorescence maxima upon excitation at different wavelengths. The values in parentheses represent the relative weight percentages of the time components.

System	λ_ex_ (nm)	λ_em_ (nm)	τ_1_ (ps)	τ_2_ (ps)	τ_3_ (ps)	τ_avg_ (ps)
Rh123	375	525	3850 (100%)			3850
Rh123-RGO	375	525	60 (52%)	3850 (48%)		1874
Rh123-Au-RGO	375	525	60 (53%)	546 (27%)	3850 (20%)	825

**Figure 8.**
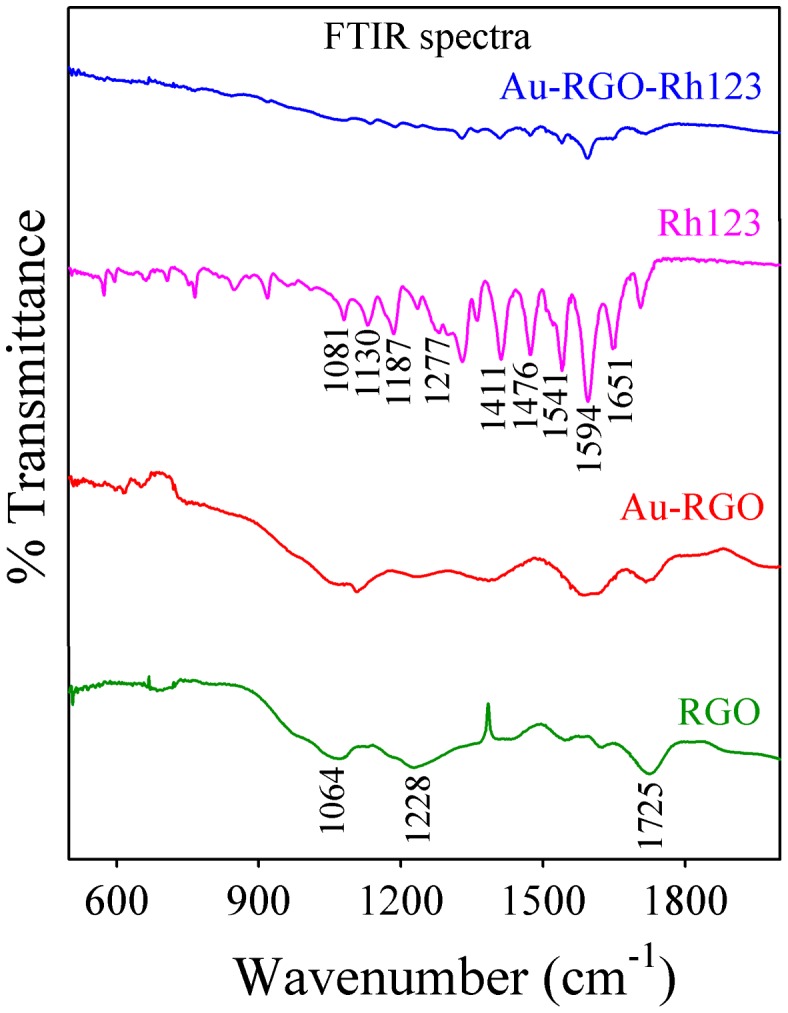
FTIR spectra of Rh123 before and after adsorption on Au-RGO.

The interaction between Rh123 and Au-RGO nanohybrid was studied by Fourier transform infrared (FTIR) absorption spectroscopy. The FTIR spectra of Rh123 before and after adsorption onto Au-RGO nanohybrid are shown in Figure 8. FTIR spectra of RGO exhibit a band at 1725 cm^–1^ corresponding to C=O stretching frequency of the –COOH group. The peak at 1228 cm^–1^ is attributed to C-OH (epoxy functional group) and the peak at 1065 cm^–1^ originates from the C-O band of the alkoxy functional group.[63] For free Rh123, the peaks at 1651, 1594, 1541, 1476, and 1411 cm^–1^ correspond to xanthene ring stretching frequencies while peaks at 1081, 1130, 187 and 1287 cm^–1^ are attributed to the C-H bending modes of the xanthene ring.[59] After adsorption of Rh123 onto Au-RGO nanohybrid, the peaks corresponding to xanthene ring and -COOH, C-OH, and C-O bands of the alkoxy group present in the Au-RGO nanohybrid are perturbed, which indicates interaction of these groups during the adsorption process. So, the -OH group of Au-RGO undergoes electrostatic interaction with = NH_2_^+^ of Rh123, which may facilitate adsorption of Rh123 onto the Au-RGO surface. Here π–π electrostatic interaction between π electrons of Rh123 and π electrons of Au-RGO nanohybrid may facilitate the adsorption of Rh123 onto Au-RGO surface, which is consistent with the reported literature.[54, 64]

**Figure 9.**
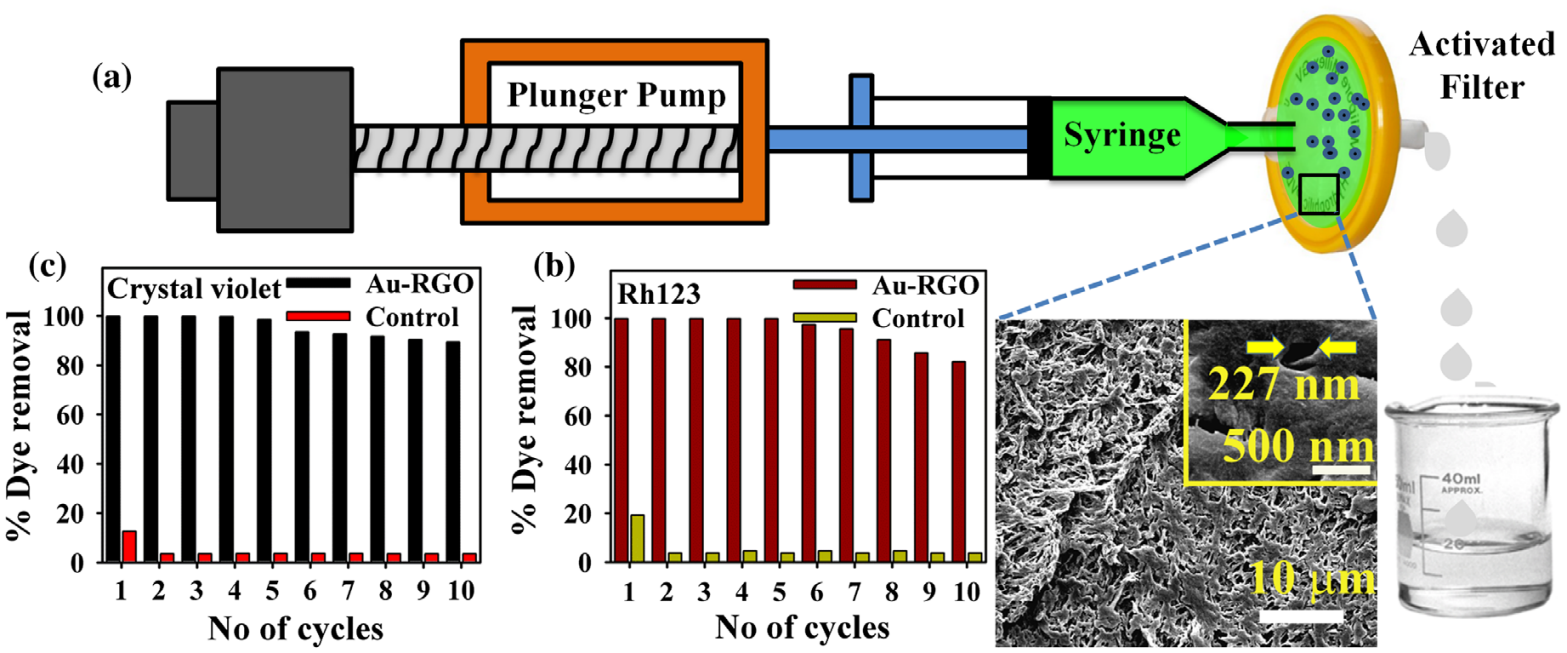
(a) Schematic representation of flow device developed for adsorption (inset shows the SEM image of filter); (b) and (c) recyclability studies of Rh123 and crystal violet respectively.

For potential application of Au-RGO nanohybrid in dye removal, we first developed a prototype device as shown in Figure 9(a). The developed device consists of 0.22 μm filter paper that is directly attached with a syringe pump. At first we loaded Au-RGO nanohybrid (10 mg) over 0.22 μm filter paper so that water can transport through the pores of the filter paper. SEM images of the filter paper shown in Figure 9(a) (the inset shows the distance between pores is approximately 227 nm). The dyes used in the adsorption process are Rh123 and crystal violet. During the adsorption process flow rate is maintained at 1 ml min^–1^ and each cycle consists of 10 ml of dye solution. From Figure 9(b) and 9(c), it is clear that Au-RGO nanohybrid is very efficient for removal of Rh123 and crystal violet, whereas under controlled conditions (absence of Au-RGO loading onto filter) adsorption of Rh123 and crystal violet is negligibly small. The results clearly demonstrate that the nanohybrid is very efficient for dye adsorption. After the 10th cycle of re-adsorption, the nanohybrid is not likely to further affect the adsorption capacity, which is almost same as the first cycle. Such a device has the advantage of easy and rapid extraction of dyes from waste water along with a recyclable feature with almost no compromise on the adsorption capacity.

## 4. Conclusions

In summary, we have developed a facile approach for the synthesis of Au-RGO nanohybrids where RGO nanosheets are decorated by a uniform distribution of Au NPs. Due to the high surface area and negative surface charge, Au-RGO nanohybrids are highly efficient for dye removal through electrostatic and π–π interactions. The efficiency of dye can be tuned by changing the pH value and the temperature of the medium. A Langmuir model has been successfully applied to show that the adsorption takes place via surface monolayer coverage. The kinetics of the dye removal process indicates that pseudo-second order kinetics are preferably followed, while thermodynamic parameters suggest that the adsorption process is spontaneous and endothermic in nature. Time-resolved fluorescence spectroscopy clarified the excited state electron and energy transfer processes associated with adsorption process. A shortening of average lifetime after adsorption of Rh123 on an Au-RGO surface leads to the NSET process because of close proximity between dye and Au NPs. Development of an engineered prototype device shows rapid efficiency as well as recyclability for dye removal from waste water. Therefore, we believe that such nanohybrids will be suitable for water purification and treatment.

## Disclosure statement

No potential conflict of interest was reported by the authors.

## Funding

This work was funded by Department of Science and Technology (DST), INDIA [grant numbers DST/TM/SERI/2k11/103; SB/S1/PC-011/2013]; and Department of Atomic Energy (DAE) (India) [grant number 2013/37P/73/BRNS].

## Supplemental data

Supplemental data for this article can be accessed here. http://dx.doi.org/10.1080/14686996.2016.1201413

## Supplementary Material

Supporting_Information.docx
