# Supplementary material for: Facile synthesis of reduced graphene oxide–gold nanohybrid for potential use in industrial waste-water treatment
Source: Sci Technol Adv Mater. 2016 Jul 26;17(1):375–86. doi: 10.1080/14686996.2016.1201413 (PMC5101912; doi:10.1080/14686996.2016.1201413)
Supplement: Supporting_Information.docx [file tsta_a_1201413_sm7821.docx]

**Supporting Information**

**Facile Synthesis of Reduced Graphene Oxide-gold Nanohybrid for Potential Use in Industrial Waste-water Treatment**

Prasenjit Kar^†^, Samim Sardar^†^, Bo Liu^§^, Monjoy Sreemany^‡^, Peter Lemmens^*§ς^, Srabanti Ghosh^*†^ and Samir Kumar Pal^*†^

**^†^***Department of Chemical, Biological and Macromolecular Sciences,*

*S. N. Bose National Centre for Basic Sciences,*

*Block JD, Sector III, SaltLake,*

*Kolkata 700 098, India*

**^‡^***Advanced Mechanical and Material Characterization Div., CSIR-Central Glass & Ceramics Research Institute, Jadavpur, Kolkata-700032, India*

**^§^***Institute for Condensed Matter Physics,*

*TU Braunschweig, Mendelssohnstraße 3,*

*38106 Braunschweig, Germany*

**^ς^***Laboratory for Emerging Nanometrology,*

*TU Braunschweig, Braunschweig, Germany*

^*^Corresponding Authors Email:

[p.lemmens@tu-bs.de](mailto:p.lemmens@tu-bs.de) (P. Lemmens),

[ghosh.srabanti@gmail.com](mailto:ghosh.srabanti@gmail.com) (S. Ghosh),

[skpal@bose.res.in](mailto:skpal@bose.res.in) (S. K. Pal)

**Adsorption models and thermodynamic parameters:**

Adsorption kinetics is an important tool in order to investigate the adsorption process. The adsorption kinetics of Rh123 on Au-RGO nanohybrid has been studied by pseudo first-order and pseudo second-order models. The Lagergren pseudo first-order kinetic model can be expressed as,

$\log(q_{e}-q_{t})=\log q_{e}-k_{1}t/2.303$, (1)

where, k_1_ is the pseudo first-order rate constant, q_e_ and q_t_ denote the adsorption capacity of Rh123 onto RGO sheet at equilibrium and at time t, respectively.

The pseudo second order kinetic model can be expressed as,

$t/q_{t}=1/k_{2}q_{e}^{2}+1/q_{e}t$, (2)

where, k_2_ is the pseudo second-order rate constant.

In order to investigate the distribution behavior of adsorbate over adsorbent Langmuir and Freundlich adsorption isotherm model are useful. Langmuir adsorption isotherm is based on the assumption that monolayer coverage of adsorbate over the adsorbent, which is assumed to be homogeneous. The Langmuir adsorption isotherm can be represented as,

$C_{e}/q_{e}=1/Q_{0}K_{L}+(1/Q_{0})C_{e}$, (3)

where, K_L_ denotes the Langmuir constant related to the energy of adsorption and Q_0_ is the Langmuir adsorption capacity. The Freundlich adsorption isotherm can be expressed as,

$\log q_{e}=\log K_{f}+(1/n)\log C_{e}$, (4)

where, q_e_ is the amount adsorbed, C_e_ is the concentration of the adsorbate, K_F_ and n are the Freundlich constants related to adsorbate and adsorbent respectively.

Furthermore, the various thermodynamic parameters associated with the adsorption process can be calculated using following equations,

$K_{d}=\frac{q_{e}}{C_{e}}$ (5)

$\Delta G^{o}=-RT\ln K_{d}$ (6)

$\ln K_{d}=\Delta S^{o}/R-\Delta H^{o}/RT$, (7)

where, K_d_ is the distribution coefficient, T is the temperature, R is the gas constant, ∆G^0^ is the change in free energy, ∆H^0^ is the change in enthalpy and ∆S^0^ is the change in entropy.
